# Accommodating quality and service improvement research within existing ethical principles

**DOI:** 10.1186/s13063-018-2724-2

**Published:** 2018-06-25

**Authors:** Cory E. Goldstein, Charles Weijer, Jamie C. Brehaut, Marion Campbell, Dean A. Fergusson, Jeremy M. Grimshaw, Karla Hemming, Austin R. Horn, Monica Taljaard

**Affiliations:** 1 Rotman Institute of Philosophy, Western University, London, Canada; 2 Clinical Epidemiology Program, Ottawa Hospital Research Institute, Ottawa, Canada; 3 Health Services Research Unit, University of Aberdeen, Aberdeen, UK; 4 Institute of Applied Health Research, University of Birmingham, Birmingham, UK

**Keywords:** Quality and service improvement research, Ethics, Informed consent, Regulation, Research ethics committees

## Abstract

**Background:**

Quality and service improvement (QSI) research employs a broad range of methods to enhance the efficiency of healthcare delivery. QSI research differs from traditional healthcare research and poses unique ethical questions. Since QSI research aims to generate knowledge to enhance quality improvement efforts, should it be considered research for regulatory purposes? Is review by a research ethics committee required? Should healthcare providers be considered research participants? If participation in QSI research entails no more than minimal risk, is consent required? The lack of consensus on answers to these questions highlights the need for ethical guidance.

**Main body:**

Three distinct approaches to classifying QSI research in accordance with existing ethical principles and regulations can be found in the literature. In the first approach, QSI research is viewed as distinct from other types of healthcare research and does not require regulation. In the second approach, QSI research falls within regulatory guidelines but is exempt from research ethics committee review. In the third approach, QSI research is deemed to be part of the learning healthcare system and, as such, is subject to a different set of ethical principles entirely. In this paper, we critically assess each of these views.

**Conclusion:**

While none of these approaches is entirely satisfactory, we argue that use of the ethical principles governing research provides the best means of addressing the numerous questions posed by QSI research.

## Background

The U.S. Institute of Medicine noted that patients routinely fail to receive high-quality, evidence-based healthcare [1]. This problem is not solely an American one, as concern over health service delivery and patient outcome is part of a global trend [2, 3]. Quality and service improvement (QSI) research seeks to redress these issues by examining different facets of the healthcare system and proposing ways to improve delivery, outcomes, and efficiency [4]. This article concerns QSI research, which are QSI activities that meet the definition of research involving human participants. This research commonly occurs in well-resourced learning health institutions with ample healthcare staff, electronic health record infrastructure, and experienced oversight committees. However, features of QSI research challenge the application of current ethics regulations. Research regulations were developed with conventional randomized controlled trials in mind where individual patients are randomized to one or more experimental interventions. QSI research differs from this paradigm in several ways: (1) QSI research typically does not involve innovative therapy but usual care; (2) it often intervenes on healthcare providers rather than patients; (3) it may involve participants at multiple levels; and (4) it is usually assumed to pose no more than minimal risk.

The Surgical Checklist Trial illustrates these differences. Post-surgical complications are an important cause of morbidity and mortality. Accordingly, in 2008, the World Health Organization designed a surgical safety checklist to improve patient outcomes [5]. The Surgical Checklist Trial evaluated the impact of this checklist on 30-day post-surgical morbidity and mortality in two Norwegian hospitals [6]. The trial employed a stepped-wedge cluster randomized design with the units of randomization being five surgical specialties. At the start of the trial, all five surgical specialties provided usual care in the operating room. The order of adoption of the 20-item checklist was randomized such that, at the end of the trial, all five specialties were using the checklist. Nurses in the operating room assessed and recorded physician compliance and patient outcome data were collected from electronic health records. The research ethics committee (REC) reviewed the trial and “advised that use of routinely collected anonymized patient data is clinical service improvement and thus no further approval or patient consent is required” [6]. The trial was approved further by the privacy ombudsman and hospital managers.

The Surgical Checklist Trial raises ethical issues common to much QSI research: should trials that aim to improve the quality of health services be considered research for regulatory purposes? When is REC review required? When the study intervention targets health providers, are they research participants? And when is health provider or patient informed consent required? Plainly, both researchers and RECs need guidance on these (and other) issues. To date, three approaches to accommodate QSI research within existing regulations have been described. In this paper, we use the Surgical Checklist Trial to illustrate these three approaches and problems associated with each. Table 1 summarizes key features of each approach, implications for the Surgical Checklist Trial, and potential problems.

**Table 1:** Current approaches to accommodating QSI research within existing research regulations

Approach	Key features	Implications for the Surgical Checklist Trial	Problems identified
QSI research is not research for regulatory purposes	For regulatory purposes, QSI research is distinct from other types of research QSI research is designed to bring about improvement in a local setting	The Surgical Checklist Trial is not research, as traditionally conceived The Surgical Checklist Trial does not require formal research ethics committee review	It is unclear how QSI research is distinct from other types of research QSI research meets the formal definition of research
QSI is research, but is exempt from REC review	QSI research often poses only minimal risk and uses routinely collected data As such, QSI research should be exempt from review	Risks of participation are minimal and the study only makes use of routinely collected and anonymized patient data The Surgical Checklist Trial is exempt from REC review	QSI research often involves study interventions such as educational programs that target human participants As such, QSI research is not exempt from REC review
QSI research is part of the learning healthcare system and requires a new ethics framework	Rejects the research-practice distinction Articulates “learning” as a novel ethical obligation QSI research that poses only minimal risk and does not infringe on patient values should be integrated into clinical care	The study poses only minimal risk and does not infringe on the rights of participants Surgeons and patients have an obligation to participate in the trial The Surgical Checklist Trial should be integrated into the learning healthcare system	Research and practice are distinct There is no compelling justification for making research participation (“learning”) an obligation

### Approach #1: QSI research is not research for regulatory purposes

Existing research regulations make a sharp distinction between research and practice [7, 8]. A clear demarcation between these two activities serves to define “the extent to which the protections required for research [participants] may differ from those required for patients” [9]. Research is defined as a class of activities designed to contribute to generalizable knowledge [10]. Practice, meanwhile, may be defined as “a class of activities designed solely to enhance the well-being of an individual patient” [10]. Differing rules therefore govern their conduct. For instance, research requires REC review, while practice does not. Research regulations codify the norms for the ethical conduct of research involving human participants.

The first approach, proposed by Lynn and colleagues, classifies QSI research as distinct from other types of research for regulatory purposes [11]. Since QSI research is designed both to improve local care and to produce generalizable knowledge, Lynn and colleagues argue that most QSI activities do not qualify as research and, consequently, do not fall within the purview of research regulations or require REC review. On their view, REC review is a “costly, cumbersome… process that is minimally relevant to” and ineffective in ensuring the ethical conduct of QSI research [11]. They suggest that the ethical oversight of QSI research should be part of an accountability system for professionals and the management of clinical care [11].

How would this first approach apply to the Surgical Checklist Trial? Recall that the goal of the trial is to improve service delivery to reduce postoperative morbidity and mortality. As the trial seeks to improve the quality of local surgical care, the trial should not be considered research for regulatory purposes.

But this conclusion is problematic. While the Surgical Checklist Trial does seek to improve care locally, it is plainly designed to contribute to generalizable knowledge. Indeed, the authors of the trial acknowledge as much in saying that its findings should be “add[ed] to [the] growing body of evidence” [6]. Further, in intervening on health providers and collecting private health information from patients, the trial involves human participants. It is precisely these two features—generalizable knowledge and the involvement of human participants—that cause other studies to be subsumed under research regulations. Thus, the Surgical Checklist Trial should be considered research for regulatory purposes.

### Approach #2: QSI research is exempt from REC review

Research involving human participants exposes individuals to risk—at least in part—for the benefit of others. REC review is designed to ensure that research conforms to regulations and, thereby, protects the liberty and welfare interests of research participants. In a restrictive set of cases, however, research involving human participants may be exempt from REC review. These exemptions mostly apply to research that has no potential impact on participants’ interests. For instance, the U.S. *Common Rule* and Canada’s *Tri-Council Policy Statement* classify research as exempt from REC review only when it involves routinely collected health information that is rigorously anonymized [12, 13].

The second approach classifies QSI research as exempt from REC review. Proponents of this view, such as Mary Ann Baily, argues that QSI research involves the sole use of routinely collected patient data that have been anonymized and are exempt from REC review [14]. A prime example of this approach is the Keystone ICU Study [15, 16]. The study involved a complex intervention, including education and a checklist, to change practitioner behavior and increase the use of evidence-based procedures known to reduce the risk of infection associated with central venous catheters. The study used routinely collected data and examined group-level infection rates. Thus, according to Bailey, “[s]uch activities should not require [REC] review” and, indeed, the REC deemed the Keystone study exempt from review [14, 16].

How would proponents of the second approach classify the Surgical Checklist Trial? The Surgical Checklist Trial used routinely collected and anonymized patient data and, according to this view, should be exempt from REC review. Indeed, this seems to have been the view of the responsible REC [6]. We disagree. The Surgical Checklist Trial should have been formally reviewed by a REC as it involved study interventions, specifically, the use of an educational program and a checklist. These interventions targeted surgeons and thereby implicated their liberty and welfare interests. As research participants, health providers are entitled to the protection of their interests afforded by REC review.

### Approach #3: QSI research is part of the learning healthcare system’s new ethical framework

The U.S. Institute of Medicine defines a learning healthcare system as one that “is designed to generate and apply the best evidence for the collaborative health care choices of each patient and provider; to drive the process of discovery as a natural outgrowth of patient care; and to ensure innovation, quality, safety and value in health care” [17].

QSI research is integral to the learning healthcare system as it provides the evidence used to improve healthcare delivery. But proponents of the learning healthcare system argue that QSI research has been hindered by “the bureaucratic burdens imposed by [REC] review processes” [4]. Reducing such burdens requires “a clear framework whether or not human [participant] research requirements apply [to QSI studies] that carry no more than minimal risk” [3].

Faden and colleagues set out a new framework to ensure that the learning activities in this system, including QSI research, are conducted ethically [18]. Their framework consists of seven obligations: (1) respect the rights and dignity of patients; (2) respect clinician judgments; (3) provide optimal clinical care to each patient; (4) avoid imposing non-clinical risks and burdens on patients; (5) address health inequalities; (6) conduct continuous learning activities that improve the quality of clinical care and healthcare systems; and (7) contribute to the common purpose of improving the quality and value of clinical care and healthcare systems.

Faden and colleagues’ framework also identifies who bears responsibility for each obligation. The responsibility for obligations (1) through (6) falls to researchers, clinicians, healthcare systems administrators, payers, and purchasers, while the responsibility for obligation (7) additionally falls to patients. Faden and colleagues also state that “each of the seven moral obligations in the framework constitutes a necessary condition…of an adequate ethics,” but that “when these [obligations] come into conflict…the goal will be to show either that one [obligation] is of overriding importance in that context or that at least some demands of each of the conflicting [obligations] can be satisfied” [18]. Thus, as we understand it, the obligations are prima facie, that is, required unless outweighed by countervailing considerations**.**

Their framework differs from standard research regulations in two ways. First, the framework stipulates that contributing to “learning” is a moral obligation for researchers, clinicians, institutions, payers, purchasers, and patients. They explain that “all involved must appreciate that they are receiving care or working in an institution committed to the shared mission of continuous learning that feeds directly into improving patient care.” [19]. Second, they deny the research–practice distinction, and argue that “different operational criteria for determining which activities should be subject to oversight policies” are needed [18]. In this proposed system, ethics oversight panels (distinct from RECs) will determine when QSI research poses only minimal risk and does not infringe on patient values [19]. Fiscella and colleagues suggest a two-step oversight process, in which a one-page document summarizing key ethical considerations is reviewed by an ethics oversight panel—constituted by investigators and previous participants of QSI research—to either approve the project or refer it to further REC review [20]. QSI research satisfying the operational criteria will be integrated into clinical care without informed consent. Institutions will notify the public that QSI research is being conducted [19].

How would this third approach apply to the Surgical Checklist Trial? Proponents would point out that both healthcare providers and patients have an ethical obligation to participate in a trial that seeks to improve health services delivery. Consultation with an oversight panel is likely to conclude that the study poses minimal risk and does not impact patient values significantly. Consequently, the Surgical Checklist Trial ought to be integrated into clinical care without seeking consent from health providers or patients, but public notification is required.

In our view, Faden and colleagues make the most persuasive argument due, in part, to their direct appeal to ethical principles. Questions remain, however, as to the adequacy of the learning health system framework. For instance, do patients in fact have an obligation to participate in QSI research? What follows from this in terms of REC review and informed consent? Might the potential for classifying practitioner behavior as substandard negatively affect their welfare interests? Finally, Faden and colleagues provide us with no details as to how the proposed framework will comply with existing regulations and statutes.

## Discussion

Our review of three different approaches to accommodating QSI research in existing regulations has found none to be wholly satisfactory. We believe that there are two reasons for this. First, attempts to address the ethics of QSI research by way of appeal to regulations are unlikely to succeed. As explained above, the patient randomized controlled trial designed for regulatory approval is the paradigm for current research regulations. QSI research departs from this paradigm; for one reason, it often involves cluster randomization to usual care or otherwise low-risk interventions that may involve participants at multiple levels. Hence, answers are unlikely to be found within such regulations. The first two approaches fail for just this reason. The first approach claims that QSI research ought not be considered research for regulatory purposes because its core objective is quality improvement. But this ignores the fact that QSI research meets the definition of research and involves human participants; in other words, the exact features that cause activities to fall within research regulations. The second approach seeks to exploit a loophole found within several regulations, namely, that research involving only routinely collected and anonymized patient data is exempt. But this ignores the fact that QSI research commonly involves study interventions directed at health providers, patients, or both, who are thus research participants.

Second, attempts to address the ethics of QSI research by way of appeal to novel ethical principles are more promising, but are likely to produce answers that conflict with existing regulations. Faden and colleagues set out a novel ethical framework for the learning health system comprising seven ethical principles, including a novel obligation for health providers and patients to participate in “learning activities.” As current regulations are grounded in differing ethical principles (that include no such obligation), conflict between a learning health system approach and existing regulations is entirely predictable [21, 22]. Moreover, as recent experience has demonstrated, effecting change to the US *Common Rule* is a lengthy and complex process that can take over a decade [23, 24].

How, then, should we proceed? We believe that direct appeal to widely accepted research ethics principles provides the best means of addressing ethical challenges in QSI research. Existing regulations were not written with QSI research in mind and are unlikely to provide solutions. But regulations are based on internationally accepted ethical principles, including respect for persons, beneficence, justice, and respect for communities [7, 9, 25]. These ethical principles articulate obligations to individuals and communities in research at a level of abstraction that confers considerable flexibility. As they serve as the ethical foundation for regulations, the potential for conflict is minimized.

What would an ethics of QSI research grounded in research ethics principles look like? Several provisional conclusions come to mind. First, the research–practice distinction is foundational to research ethics principles. When a QSI activity meets the definition of research and involves human participants, it must be reviewed by an REC. But if there is substantial doubt as to whether an activity meets these definitions, it is incumbent on the investigators to ask the REC to provide a written determination as to whether the activity requires review in advance of initiating the activity. We believe that this position is both ethically required and prudent, as journals are requiring evidence of REC review for QSI research [26]. However, when it is clear that a QSI activity does not have human participants or does not meet the definition for research, it does not need REC review.

Second, participants in QSI research must be clearly identified, so that their interests may be protected. QSI research commonly involves study interventions that target health providers and, in our experience, researchers and RECs may fail to recognize this fact. Health providers who serve as research participants are a vulnerable population in part due to their susceptibility to coercion in the work environment and are therefore entitled to protection. Resnik offers several strategies for protecting employees in research; for example: (1) research proposals should describe measures to protect the rights and welfare of employees; (2) supervisors should not directly recruit employees and their interactions should be restricted; (3) the informed consent process should clearly indicate participation will not impact employment status or benefits and an independent party should monitor the process [27].

Third, the multi-level and complex nature of QSI research has implications for the role of informed consent. Investigators conducting QSI research may use cluster randomized designs, in which the unit of allocation, study interventions, and outcome measurement may differ. The *Ottawa Statement for the ethical design and conduct of cluster randomized trials* provides helpful guidance for obtaining informed consent in such designs [28]. First, when study interventions and data collection procedures target different individuals, informed consent should be obtained from research participants for different aspects of the study relevant to their participation. Second, researchers should identify participants and seek their informed consent before cluster randomization if possible; if not possible, their informed consent should be obtained as soon as possible and prior to any study intervention or data collection procedure. Finally, a waiver of consent may be appropriate when participants cannot reasonably avoid a health system intervention that poses only minimal risk.

Fourth, RECs should use a proportionate approach to the review of QSI research. Studies that pose substantial risk or involve vulnerable participants ought to receive intensive scrutiny; QSI research that poses low risk and does not involve vulnerable participants may undergo expedited review. For example, the ARECCI (A pRoject Ethics Community Consensus Initiative) Ethics Screening Tool has been implemented in Alberta Canada [29]. It is premised on the idea that all QSI activities raise ethical issues, where some activities require full REC review and others require delegated review. The ARECCI tool separates research and non-research activities through an electronic questionnaire that helps determine the primary purpose of the project, the types of ethical risks and the appropriate type of ethics review [29]. Plainly, though, a comprehensive answer to this question requires further work.

## Conclusion

Researchers and RECs need comprehensive guidance in the interpretation and application of accepted ethical principles for QSI research. Taking into consideration the three well-intentioned, but ultimately unsuccessful, attempts to accommodate QSI research within existing ethical principles and regulation, the way forward is to use established ethical principles. We draw tentative conclusions on four issues: there is a morally relevant distinction between research and practice; participants of QSI research need to be clearly identified and protected; informed consent ought to be obtained, when possible, for relevant aspects of the QSI research; and QSI research should undergo proportionate review by RECs.

## Abbreviations

ICU: Intensive care unit
QSI: Quality and service improvement
REC: Research ethics committee
